# Relative difference among 27 functional measures in patients with knee osteoarthritis: an exploratory cross-sectional case-control study

**DOI:** 10.1186/s12891-019-2845-0

**Published:** 2019-10-22

**Authors:** K. Vårbakken, H. Lorås, K. G. Nilsson, M. Engdal, A. K. Stensdotter

**Affiliations:** 1 Department of Neuromedicine and Movement Science, Norwegian University of Science and Technology, Trondheim, Norway; 2 Faculty of Medicine and Health Sciences, NTNU, Health og Social building, 7491 Trondheim, Norway; 3 Department of Physical Education and Sport Science, Nord University, Levanger, Norway; 4 Surgical and Perioperative Sciences, Umea University, Umea, Sweden; 5 Department of Physiotherapy, Clinic of Clinical Services, Trondheim University Hospital, Trondheim, Norway

**Keywords:** Osteoarthritis, knee, Muscle strength dynamometer, Physical fitness, Physical examination, Physical activity, Exercise, Activities of daily living, International classification of functioning, disability, and health, Psychology, Sociological factors

## Abstract

**Background:**

To raise the effectiveness of interventions, clinicians should evaluate important biopsychosocial aspects of the patient’s situation. There is limited knowledge of which factors according to the International Classification of Function, Disability, and Health (ICF) are most deviant between patients with knee osteoarthritis (KOA) and healthy individuals. To assist in measures’ selection, we aimed to quantify the differences between patients with KOA and healthy controls on various measures across the ICF dimensions of body function, activity, and participation.

**Methods:**

We performed an exploratory cross-sectional case-control study. In total, 28 patients with mild-to-moderate KOA (mean age 61 years, 64% women) referred by general physicians to a hospital’s osteoarthritis-school, and 31 healthy participants (mean age 55 years, 52% women), volunteered. We compared between-group differences on 27 physical and self-reported measures derived from treatment guidelines, trial recommendations, and trial/outcome reviews. Independent t-test, Chi-square, and Mann-Whitney U test evaluated the significance for continuous parametric, dichotomous, and ordinal data, respectively. For parametric data, effect sizes were calculated as Cohen’s *d*. For non-parametric data, *d*s were estimated by *p*-values and sample sizes according to statistical formulas. Finally, all *d*s were ranked and interpreted after Hopkins’ scale. An age-adjusted sensitivity-analysis on parametric data validated those conclusions.

**Results:**

Very large differences between patients and controls were found on the Pain numeric rating scale^1^, the Knee Injury and Osteoarthritis Scale (KOOS, all subscales)^2^, as well as the Örebro Musculoskeletal psychosocial scale^3^ (*P* < 0.0001). Large differences were found on the Timed 10-steps-up-and-down stair climb test^4^ and Accelerometer registered vigorous-intensity physical activity in daily life^5^ (*P* < 0.001). Respectively, these measures clustered on ICF as follows: ^1^body function, ^2^all three ICF-dimensions, ^3^body function and participation, ^4^activity, and ^5^participation.

**Limitations:**

The limited sample excluded elderly patients with severe obesity.

**Conclusions:**

Very large differences across all ICF dimensions were indicated for the KOOS and Örebro questionnaires together for patients aged 45–70 with KOA. Clinicians are suggested to use them as means of selecting supplementary measures with appropriate discriminative characteristics and clear links to effective therapy. Confirmative studies are needed to further validate these explorative and partly age-unadjusted conclusions.

## Background

Osteoarthritis is the second most prevalent condition of all musculoskeletal and rheumatic diseases and the main contributor to social activity limitations [1, 2]. Knee osteoarthritis (KOA) has an incidence of 240 per 100,000 person-years in adults, which is more than 2.5 times higher compared to osteoarthritis of the hip [3].

Traditionally, KOA has been diagnosed by radiography and arthroplasty considered to be the only effective treatment [4]. Recent governmental-approved guidelines for primary care in Denmark and Sweden, however, state that a KOA diagnosis can be made clinically and that the first-line of care is physiotherapy-guided training and education [5]. This is supported through the diagnostic criteria provided by the European League Against Rheumatism (EULAR), developed specifically for primary care [6]. According to EULAR, clinical examination alone can offer a confident diagnosis of KOA. Other diagnostic criteria such as from the American College of Rheumatology (ACR) [7], are primarily developed for hospital care [6].

KOA has long been recognized as a whole *organ* disease [4], but has more recently been explained as a whole *person* chronic disease [4]. By the latter understanding, symptoms and signs most often develop slowly over decades [8] and can be manageable for most people through an early diagnosis and individualized strategies [4]. This new paradigm requires a holistic view on diagnosis, clearly linked to self-management aiming to improve the patient’s prognosis in the long term.

In view of the International Classification of Function, Disability, and Health [ICF] [9], which represents a systems theory and biopsychosocial understanding [4, 10], the diagnostic criteria provided by EULAR and ACR offers limited understanding of the overall clinical situation in patients with KOA. In particular, the criteria do not consider the ICF dimensions *activity* and *participation*. Furthermore, similar limitations seem to apply to known risk and prognostic factors documented in systematic reviews [11–13] in which the predominant factors evaluated are summarised mainly into body functions, personal, and disease-related factors.

Acknowledging KOA as a whole person disease requires a holistic and biopsychosocial approach. A proper diagnostic assessment needs to include factors derived across the ICF dimensions in order to pinpoint the most important measures for empowering patients to self-manage and cope with their most valued functional goals [14, 15]. In a level 1 study of diagnostics, the relevant question is to ask what assessments clearly differ between patients and healthy controls [16]. As indicated by available diagnostic guidelines (EULAR, ACR), as well as evidence from systematic reviews (referred above), that discrimination question has gained little attention in KOA viewed from an overall ICF perspective.

Thus, the main objective of the current study was to explore between-group differences in individuals with KOA and healthy controls, by applying a battery of functional measures derived from guidelines, trial recommendations and previous systematic reviews [17–20] that captures a spectrum of ICF dimensions. The second objective was to examine the rank of these between-group differences (by effect sizes) in order to pinpoint the most deviant functions. The third objective was to analyse how these measures cluster on the ICF dimensions.

## Methods

### Design and ethics

We aimed to perform a cross-sectional, explorative, matched case-control study. The study was approved by the Regional Ethics Committee for Medical and Health Research (REC 2016/984) and was conducted according to the Helsinki declaration. All participants received oral and written information and signed an informed consent form before entering the study. Based on a moderate effect size in knee extension strength between similar groups, i.e. applying unpublished data from our lab related to an earlier study [21], our a priori sample size calculation indicated that we required 20 participants in each group, as the study was allocated 80% power to detect an effect at *p* < 0.05 (cf. Statistics analysis). Although we assumed that no adjustments were needed for multiple comparisons in exploratory studies [22–27], we still aimed for 30 participants in each group. We recruited individuals with KOA referred by general physicians (GPs) to private physiotherapy clinics and to the osteoarthritis school at Trondheim University Hospital, from Nov 2016 to Dec 2017. In about the same period, healthy volunteers were recruited via job visits, posters, flyers, and electronic communication, from several different work places in the vicinity of the lab.

Data for each individual participant were collected within a period of approximately 2 weeks through questionnaires and functional tests in the lab. The questionnaires were e-mailed as web-surveys together with the informed consent forms through the Infopad system [28]. The groups were aimed to be frequency-matched [23] on age and gender through the eligibility criteria below. The study was extensive as the questionnaires took on average 40 min and the physical functional test protocol on average 2.7 h in the lab. (The current paper presents data from a larger study.) At the very end of the lab session, an accelerometer sensor was applied on the anterior left thigh [29, 30] and worn for all hours during 1 week, before it was returned by mail.

### Participants eligibility

The inclusion criteria for patients were having KOA in the tibiofemoral joint(s) diagnosed clinically (by GPs or physiotherapists) and radiologically [31], main problem of pain and limited physical function related to the knee(s), be symptomatic for > 3 months and daily in the last month, understand Norwegian (orally and written), and be within 45–70 years old. Both genders were included. The upper age-limit was sat to prevent possible confounding due to between-group differences in (i) comorbidity [32] and (ii) physical activity (due to retirement/freedom to reduce activity according to KOA-symptoms), as well as an decline in body function naturally exhibited at high age in both groups [33].

The inclusion criteria for healthy volunteers were aimed to be age and sex frequency-matched to the patients, and able to walk on even ground and negotiate stairs without pain and having no knee complaints.

The exclusion criteria for all participants were surgery to a lower extremity < 3 years ago, prior lower limb fractures, generalized pain, pain from the spine, hips, or ankles competing with that from the knee, body mass index (BMI) > 35 [for repeatable optokinematic recordings [34] in the main study, data not reported here], and medical diagnoses other than KOA with clear negative influence on physical function and pain.

### Measurements

The following health status constructs and instruments were implemented, building on prior recommendations and evidence [17–20] and sorted on ICF dimensions. That is, we mainly applied recommended measures from the 2010 Dutch physiotherapy guideline for patients with knee and hip osteoarthritis [17], the Osteoarthritis Research Society International (ORASI) Clinical Trial Recommendations [18], a systematic review on performance-based measures in KOA [19], and measures applied in randomized controlled trials (RCTs) reviewed according to the Cochrane Handbook [20]. Some instruments cross ICF dimensions and are thus presented shortly under more than one ICF dimension. Additional file 1 gives a detailed overview of the measurement properties and scores-of-interpretation of all the applied instruments. Below we present them briefly.

### Measures across all the ICF dimensions: body function, activity, and participation

The Knee Injury and Osteoarthritis Outcome Scale (KOOS), is a freely available knee-specific, self-reporting outcome measure (SROM) for knee-related problems [35]. It measures Pain, Symptoms, Activities in daily living (ADL), Sport and recreation (Sports/Rec), and knee-related quality of life (QoL) [36, 37]. The scores are converted to percentages, 0 to 100, worst to best. KOOS includes the Western Ontario and McMaster Universities Osteoarthritis Index (WOMAC) 3.0 to ensure validity for older individuals.

### Measures on the ICF body function dimension

On body function we used 14 measures. Four were mainly performance-based: the Biodex System 4 dynamometer [38, 39] [Biodex System Pro™, Biodex Medical Systems, NY, USA] for knee extension strength (see Procedures below); the six-minute walk distance test [6MWT] [40] for aerobic endurance; the 30 s Chair to Stand Test (n30sCST) [*n* = number of stands] [40] according to Osteoarthritis Research Society International’s (OARSI) video-descriptions [41] for fitness; and the Timed maximum 30 s single-leg stance [T30sSLS] [42] for balance.

Six were pure SROMs: the Numeric Pain Rating Scale (NPRS) [43] for unidimensional pain; two custom-made SROMs for Sleep problems and Vitality (Additional file 1); the KOOS-Pain [44] and -Symptoms [44]; and Tampa Scale of Kinesiophobia (TSK-13) [45] for fear of motion or re-injury. Finally, four were SROMs post performance tests [46]: Borg’s Rating of Perceived Exertion Category Ratio 10 (RPE-CR10) and NPRS, both post the 6MWT and the n30sCST.

### Measures on the ICF activity dimension

We used nine measures on the activity dimension. Three were performance-based: the Timed 10-step up-and-down stair climb test [T10StUpDw] [47], the Timed up-and-go [TUG] [40] for mobility, balance, walking ability, and fall risk [35], and the 6MWT [47] for long-term walking ability. Three SROMs: KOOS-ADL and -Sports/Rec for activity problems, and the Patient specific functional scale [PSFS] [48] for the three most problematic activities. Finally, three measures were SROM: the NPRS given directly after the T10StUpDw, 6MWT, and the TUG.

### Measures on the ICF participation dimension

We used four measures on the participation dimension. KOOS-QoL for knee-related quality of life [36, 37], the European Health Interview Survey-Quality of Life 8-item index [EUROHIS-QoL] [49] for generic QoL, the Örebro Musculoskeletal Pain Screening Questionnaire 10-items [OMSPQ-10] [50] for work and psychosocial factors, and the AX3 3D accelerometer [3-axis logging accelerometer, Axivity Ltd., Newcastle, UK] [29, 51] for time in four intensities of physical activities in daily life (PADL).

### Procedures

Before the lab-session, all participants filled out their respective relevant SROMs. Only the patients, however, registered PSFS and TSK-13. In the lab, within approximately 1 week after the questionnaires, we registered participants’ characteristics, degree of radiographic KOA (radiology reports), T30sSLS (on two force plates, Type 9260AA, Kistler, NY, USA), TUG, n30CST, 6MWT, and T10-StUpDw. At the end of the lab session, we measured peak strength (detailed below).

Strength (peak torque) was recorded concentrically for the quadriceps muscle at 60°/s of five maximal repetitions applying the passive concentric isokinetic mode [52] of the Biodex® System 4. The participants warmed up by the performance tests 6MWT and 10StUpDw, and a set of 15 repetitions at low-moderate load (in knee extension-flexion). Strength tests were performed with 70° trunk inclination, all starting with the right side before the left according to the Biodex manual [52]. The passive mode was chosen due to its feasibility in eccentric mode (data not reported here). Fully passive recordings were taken in order to correct for gravity (see Data processing). The system was calibrated before each session. At the very end of the lab-session, an accelerometer was fixed to the participants’ left thigh for recording of PADL at 100 Hz for 1 week.

### Data processing

For T30sSLS data, the time on one leg from the force plates was analyzed using a custom-made algorithm based on inflections in MatLab (v. R2016a, MathWorks Inc., USA). Participants stood with one leg on each force plate and the time on one leg was recorded when a foot left one of the plates. All outputs were validated by inspecting the force graphs using Qualisys Track Manager (QTM, Qualisys AB, Sweden), because the algorithm was invalid for a few recordings where participants stood on one leg already before the start of data registration.

The AX3 accelerometer data was categorized into four different intensities of PADL applying the OmGui Software [53]. Additional file 1 describes the cut-points for the four PADL-intensity levels.

For knee extensor strength, the passive torques were added to the active ones in order to correct for the limb’s own torque. This was done at the 30° knee-flexed position (0° was the straight knee) in order to minimize the passive length-tension influence. The result was calculated as best of five repetitions divided by body weight and reported at the 30° knee flexed position.

### Process: recruitment change, age-mismatch, and sensitivity analysis

Insufficient recruitment from primary physiotherapy care, older patients (able to be) recruited from hospital care, and low recruitment of older healthy volunteers, inflicted a breach in the age-matching. The age-difference necessitated an age-adjusted statistical sensitivity analysis of the parametric data (cf. Statistics below).

### Statistics analysis

Our sample size calculation assumed that no adjustments were needed for multiple comparisons in an exploratory study [22–27] and was performed in the recognized [54] freeware G*Power version 3.1.9.1 [55–57]. This power analysis was an a priori required sample size computation based on t-test (the difference between two independent means), with the following factors: Two tails, α error probability = 0.05, β error probability = 0.2 (i.e., power 1 - β = 0.8 or 80%), and a moderate effect sized [58] Cohen’s *d* = 0.914 for concentric isokinetic knee extension strength at 60°/s. This required 20 participants in each group. (Additional output parameters were: noncentrality parameter δ = 2.891, critical t = 2.024, degree of freedom = 38, total sample-size 40, actual power = 0.804).

For continuous data, normality was inferred by histogram inspections, quantile-quantile plots, and Kolmogorov-Smirnov tests. For the equal variance assumption, Levene’s test was performed. Then, for parametric data with no significant outliers and equal variance, standardized mean difference (SMD) by Cohen’s *d* was calculated with 95% CI. Independent t-tests were also performed (SPSS v.24, IBM, NY, USA). For continuous non-parametric data, we calculated median differences and 95% CI (StatsDirect v.2.8.0, Statsdirect Ltd., Cambridge, UK). Then we tested the latter data for differences between groups by medians using Mann-Whitney U (SPSS), before the U statistics and sample size were used to point-estimate Cohen’s *d* applying validated formulas [59, 60] at psychometrica.de [61].

Differences between groups on categorical variables were compared by Chi-square test for gender and the Mann-Whitney U test for education and sleep. Then, point-estimates of Cohen’s d were calculated via χ2 and U statistics, and sample sizes [59–61]. The alpha-level was set to 0.05 for the two-sided tests [22, 24, 25]*.*

Data which pertained to both groups, were then compiled and ranked on point-estimates of SMD. The size of the SMD was interpreted according to Hopkins [58] and *p*-value values according to Rosner [62] (Additional file 1). Due to the statistically significant age-difference (see Results), we performed a sensitivity analysis of co-variance (ANCOVA) with age as the covariate on the parametric data.

### Qualitative analyses

For measures with at least moderate effect size, the clustering on ICF dimensions of (*i*) body function, (*ii*) activity, and (*iii*) participation were based on a content analysis according to the ICF manual [9], definitions of activity as “the ability to move around” [63], and of participation as “the ability to perform daily activities” [64].

## Results

### Flow of participants and centres

Two participants were recruited in physiotherapy clinics, without information on those who declined. At the hospital, we recruited 26 patients out of 36 eligible, where 10 of those invited declined to participate motivated by long traveling distances (*n* = 3), not interested (*n* = 4), afraid of strength testing (*n* = 2), and too time-consuming (*n* = 1). One participant answered the questionnaire but withdrew from the study before the lab-test due to a flare-up and was excluded from the analysis. Five individuals did not qualify for participation due to old age (*n* = 3), BMI, and an unstable heart. The 31 healthy control individuals who volunteered represented academic (*n* = 10), administrative (*n* = 6), and health care personnel (*n* = 7). Further, salespersons (n = 3), industry employees (n = 3), and canteen staff (*n* = 2).

### Participant characteristics

The patients with KOA were on average 6.4 years older. There were no other significant differences on personal factors. On average the patients had had pain for 11 years, had been diagnosed 10 years ago, and showed mostly small-to-moderate radiographic KOA (Table 1).

**Table 1:** Personal and health characteristics in the case- and control group

ICF	Variables	Cases (*n* = 28)	Controls (*n* = 31)	M or Med diff	M or Med d or WG 95% CI	Statistics t, χ2, U	*P*-value
Personal factors	Female, n (%)	18 (64)	16 (52)			379 (χ2)	0.3294
	Age, yrs., M (SD)	61.7 (6.4)	55.3 (8.0)	6.4	2.6, 10.2	3.4 (t)	0.0014†
	Height, m, M (SD)	1.72 (0.10)	1.73 (0.09)	−0.02	−0.07, 0.03	− 0.7 (t)	0.517
	Weight, kg, M (SD)	82.9 (12.7)	80.4 (16.6)	2.5	−5.2, 10.3	0.7 (t)	0.517
	BMI, kg/m^2^, M (SD)	24.3 (3.5)	25.2 (5.1)	1.1	−1.1, 3.3	1.0 (t)	0.317
	Education, n (%)						
	secondary school (10 yrs)	1 (4)	0 (0)				
	high school (13 yrs)	6 (21)	6 (19)				
	graduate (16 yrs)	14 (50)	13 (42)				
	post graduate (18 yrs. +)	7 (25)	12 (39)			368 (U)	0.281
	Dominant leg (right, left, n)	26, 2	28, 3				
Patients’ body function & structure & activity factors	Yrs since diagnosis, M (SD)	10.2 (8.6)			6.9, 13.6		
	Yrs of knee pain, n (%)						
	1 yrs	2 (7)					
	1 to 3 yrs.	3 (11)					
	3 to 10 yrs	7 (25)					
	> 10 yrs	16 (57)					
	Affected knee (n, %)						
	One	14 (50)					
	Both	14 (50)					
	Pain medication (n, %)						
	None	15 (54)					
	Paracetamol	5 (18)					
	NSAIDs	5 (18)					
	Opoids	2 (7)					
	Others	1 (4)					
	TSK Fear of mov., M (SD)	24.4 (7.7)			21.4, 27.4		
	PSFS Activity 1, Med (IQR)	3.0 (5.0)			1.0, 5.0		
		Case-group only					
	X-ray grade (n knees, %)	Inv. leg	Uninv. leg				
	No X-rays taken	0 (0)	10 (36)				
	KL-grade II	9 (32)	9 (32)				
	KL-grade III	17 (61)	8 (29)				
	KL-grade IV	2 (7)	1 (4)				

**Notes.** Case-group = patients with knee osteoarthritis; control-group = individuals without knee complaints; ICF = the International Classification of Function, Disability, and Health of the WHO; M = mean; Med = median; diff = difference between groups; d = 95% difference between groups; WG = 95% difference within a group; BMI = body mass index; NSAIDs = Non-Steroid Anti-Inflammatory Drugs; TSK = Tampa Scale of Kinesiophobia; mov. = movement/reinjury; PSFS = Patient-Specific Functional Scale questionnaire; KL = Kellgren-Lawrence Grade, † = highly significant result

### Main results

The ANCOVA sensitivity analysis indicated only small bi-directional changes in *p*-values after adjustment for age (Table 2) Thus, we refer to the results of the main analysis below.

**Table 2:** Sensitivity analysis comparing mean values adjusted for age by an ANCOVA to that of the unadjusted mean values by the Independent t-test. Only the variable Moderate-intensive physical activity in daily life (registered by accelerometer) reduced its effect size one level due to the adjusted analysis. The *P*-values changed in both directions

ICF	Variables	Cases (n = 28)		Control (n = 31)		Unadj M d %	Adj M d %	D %, U - A	ES Eta^2^	ES inter-pret.	P	P
		Unadj M (SD)	Adj M (SE)	Unadj M (SD)	Adj M (SE)						Unadj	Adj
Body Function Level	30sCST, n	14.0 (4.2)	14.2 (0.8)	16.3 (4.2)	16.2 (0.8)	15.2	13.2	2	0.049	Small	0.042*	0.096
	RPE-6MWT	6.0 (1.8)	6.1 (0.3)	5.5 (1.7)	5.4 (0.3)	8.7	12.2	−3.5	0.039	Small	0.257	0.139
	RPE-10StUpDw	3.3 (1.7)	3.4 (0.3)	3.1 (1.3)	3.1 (0.3)	6.3	9.2	−2.9	0.011	Small	0.546	0.427
	Knee ext strength inv. leg, Nm/kg	1.16 (0.48)	1.15 (0.09)	1.46 (0.38)	1.48 (0.08)	22.9	25.1	−2.2	0.108	Mod.	0.010*	0.012*
	Knee ext strength uninv. Leg, Nm/kg	1.45 (0.36)	1.45 (0.08)	1.64 (0.46)	1.64 (0.08)	12.3	12.3	0.0	0.043	Small	0.088	0.12
Activity Level	10StUpDwT, sec	10.8 (3.1)	10.4 (0.4)	7.8 (1.1)	8.1 (0.4)	32.2	24.9	7.3	0.198	Large	3.6E-5‡	4.7E-04†
	TUG, sec	6.6 (1.2)	6.5 (0.2)	5.7 (0.9)	5.8 (0.2)	14.6	11.4	2.2	0.84	Mod.	0.0014†	0.027*
	6MWT, m	642.5 (94.6)	651.5 (16.6)	717.4 (75.4)	709.2 (15.7)	11.0	8.4	2.6	0.094	Mod.	0.0014†	0.019*
Particip.	Örebo PsySoc	39.0 (12.7)	38.0 (2.2)	12.8 (9.6)	13.7 (2.1)	101.2	93.0	8.2	0.515	V. L.	1.6E-12§	2.3E-10§
	Mod.-int. act., min/wk	286.0 (169.1)	306.6 (29.1)	382.5 (134.5)	363.7 (27.5)	28.9	17.0	11.9	0.032	Small*	0.018*	0.17

**Notes.** Mod.-int. act. = moderate-intensive physical activity in daily life, the only variable which showed a one level downward adjustment on effect size due to the adjusted analysis; Unadj. M d % = Unadjusted mean difference in per cent; D % U-A = difference in per cent Unadjusted mean % difference minus Adjusted mean % difference; P = the exact P-value; 30sCST = number of chair-to-stand raise in 30 s; RPE-6MWT = rate of perceived exertion at the end of the six-minute walk test (scaled 0–10, no exertion to highest exertion); RPE-10StUpDw = rate of perceived exertion at the end of the 10 step up and down stair climb test; Knee ext. strength inv. leg = Knee extension strength test on the involved leg and the uninvolved leg are given at the 30° flexed knee position (straight knee is 0°); 6MWT = the distance (in m) walked during the six-minute walk test; Örebro PsySoc = the Örebro Musculoskeletal Psychosocial Questionnaire 10 items, a 1 to 100 scale, best to worst. ES Eta^2^ = an effect size estimate equal to partial η^2^ scaled > 0.01 = small, > 0.06 = moderate, > 0.13 = large. * = significant result, † = highly significant result, ‡ = very highly significant result, § = extremely highly significant result

**Fig. 1:**
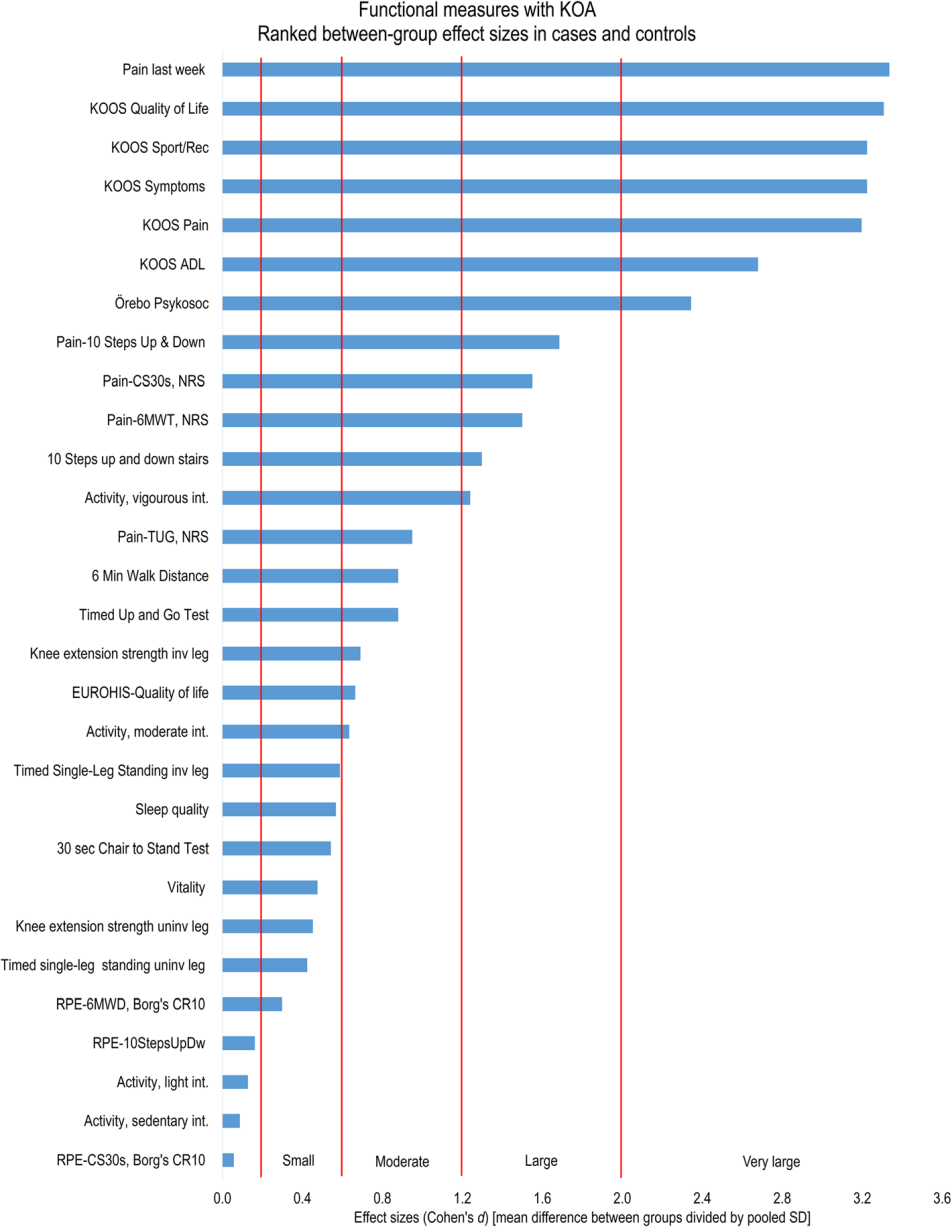
Relative difference among functional measures in patients with knee osteoarthritis compared to individuals without knee complaints. [Cf. Table 3 and the Result section text for how these measures cluster on the ICF dimension(s)]

Highly significant group differences and very large effect sizes were found for (i) body function on Pain last week, KOOS-Symptoms, and KOOS-Pain; for (ii) activity on KOOS-Sport/Rec and KOOS-ADL; and for (iii) participation on KOOS-QoL and Örebro. Table 3 shows the statistical details and Fig. 1 an overview of the rated effect sizes.

**Table 3:** Relative difference in functional measures between the case- and control-group within each ICF-dimension

ICF	Variables	Cases (n = 28)	Controls (n = 31)	ES or Med d	ES or Med d 95% CI	Stats t, χ2, U, M	P	E Abs ES or C’s *d*
Body Function	Pain last wk., Med (IQR) (R)	3.5 (4.8)	0.0 (1.0)	3.0	3.0, 5.0	0.000 (U)	2.0E-12§	3.3
	KOOS Pain, Med (IQR)	58.8 (18.8)	98.4 (3.6)	−38.9	−52.8, −30.6	5.0 (U)	1.8E-11§	3.2
	KOOS Symptoms, Med (IQR)	58.9 (33.9)	98.4 (3.6)	−35.8	−42.9, −28.6	4.0 (U)	4.6E-11§	3.2
	Pain-10StUpDw, Med (IQR) (R)	2.0 (3.0)	0.0 (3.0)	2.0	1.0, 3.0	108 (U)	1.7E-08§	1.7
	Pain-30sCST, Med (IQR)	2.0 (5.0)	0.0 (0.1)	2.0	1.0, 4.0	124 (U)	2.4E-08§	1.6
	Pain-6MWT, Med (IQR) (R)	3.5 (6.8)	0.0 (3.0)	2.0	0.0, 5.0	130 (U)	0.00061‡	1.5
	Pain-TUG, Med (IQR)	0.1 (2.7)	0.0 (0.1)	0.2	0.0, 1.0	217 (U)	1.0E-5‡	0.9
	Knee ext. strength inv. leg, M (SD)	1.16 (0.48)	1.46 (0.38)	−0.7	−0.2, −1.2	1.592 (t)	0.010*	0.7
	30sTSLS inv. leg (s), Med (IQR)	11.0 (25.4)	29.8 (7.3)	−9.1	−21.1, 0.0	292 (U)	0.0291*	0.6
	30sCST (n), M (SD)	14.0 (4.2)	16.3 (4.2)	−0.5	− 1.1, 0.0	−2.1 (t)	0.0422	0.5
	Knee ext. stren., uninv. leg, M (SD)	1.45 (0.36)	1.64 (0.46)	−0.5	−1.0, 0.1	− 1.74 (t)	0.088	0.5
	TSLS uninv leg (s), Med (IQR)	15.9 (23.5)	29.5 (16.0)	−5.4	−14.1, 0.0	329 (U)	0.1071	0.4
	RPE-6MWT, M (SD)	6.0 (1.8)	5.5 (1.7)	0.3	−0.2, 0.8	1.1 (t)	0.2577	0.3
	RPE-10StUpDwT, M (SD)	3.3 (1.7)	3.1 (1.3)	0.2	−0.3, 0.7	0.6 (t)	0.5368	0.2
	RPE-30sCST, Med (IQR)	3.0 (3.0)	3.0 (2.0)	0.0	−1.0, 1.0	420 (U)	0.8266	0.1
Activity	KOOS Sport/Rec, Med (IQR)	30.0 (25.6)	100 (5.0)	−65.0	−75.0, −60.0	4.0 (U)	2.3E-11§	3.2
	KOOS ADL, Med (IQR) (R)	67.7 (39.6)	100.0 (13.9)	−32.4	−38.2, −14.7	28.5 (U)	9.5E-11§	2.7
	10StUpDwT (s), M (SD)	10.8 (3.1)	7.8 (1.1)	1.3	0.7, 1.9	5.0 (t)	3.6E-05‡	1.3
	Timed Up-and-Go (s), M (SD)	6.6 (1.2)	5.7 (0.9)	0.9	0.3, 1.4	3.4 (t)	0.0014†	0.9
	6MWT (m), M (SD)	642.5 (94.6)	717.4 (75.4)	−0.9	−1.4, −0.3	−3.4 (t)	0.0014†	0.9
Participation	KOOS QoL, Med (IQR) (R)	43.8 (25.0)	100.0 (25.0)	−56.2	−62.5, −43.8	1.0 (U)	9.8E-12§	3.3
	Örebo PsySoc, M (SD)	39.0 (12.7)	12.8 (9.6)	2.3	1.7, 3.0	9.0 (t)	1.6E-12§	2.3
	Vig.-int. act. (min/wk), Med (IQR)	1.0 (15.3)	29.0 (63.0)	−23.0	−46.0, −10	167.5 (U)	4.5E-05‡	1.2
	EUROHIS-QoL, Med (IQR)	33.0 (4.4)	36.0 (6.0)	−3.0	−5.0, −1.0	274.5 (U)	0.015*	0.7
	Mod.-int. act. (min/wk), M (SD)	286.0 (169.1)	382.5 (134.5)	−0.6	−1.2, −0.1	−2.4 (t)	0.018*	0.6
	Light-int. act. M (SD)	1553.0 (444.9)	1494.9 (468.6)	0.1	−0.4, 0.6	0.5 (t)	0.63	0.1
	Sed.-int. act. (min/wk); M (SD)	8141.9 (663.1)	8197.5 (662.9)	−0.1	−0.6, 0.4	−0.3 (t)	0.74	0.1

**Notes.** Case = patients with knee osteoarthritis, control = individuals without knee complaints; ICF = = the International Classification of Function, Disability, and Health (of the WHO); ES = effect size by Cohen’s d or mean difference between groups divided by the variability of groups; Med d = median difference; CI = confidence interval; Stats = statistics; P = probability value; E = estimated; Abs = absolute value; ES = effect size or Cohen’s d; C’s d = Cohen’s d.; KOOS = Knee Injury and Osteoarthritis Outcome Scale; Med = median; IQR = interquartile range; R = range (given for the controls when the IQR was not obtainable); 10StUpDw = 10 step up-and-down stair climb test; 30sCST = number of Chair-to-Stand raises in 30 s test; 6MWT = test of distance walked in 6 minutes; TUG = Time up-and-go test or seconds taken to rise from a chair, walk 3 m, turn, walk back, and sit down; 30sTSLS inv. leg = 30 s timed single-leg standing on the involved leg; RPE = Rate of Perceived exertion on the Borg Category Rating Scale; Sports/Rec = sports and recreation scale; ADL = activity of daily life; QoL = Quality of Life; PsycSoc. = psychosocial questionnaire; Vig.-int. Act. = Vigorous-intensity physical activity of daily life; Mod. = moderate; Sed. = sedentary; * = significant result, † = highly significant result, ‡ = very highly significant result, § = extremely highly significant result

Highly significant group differences and large effect sizes were found for (i) body function post performance on pain-10StepUpDw, pain-CS30s, and pain-6MWT; for (ii) activity on 10StepUpDw; and for (iii) participation on vigorous-intensity PADL (Fig. 1, Table 3).

Significant group differences and moderate effect sizes were found for (i) body function on pain-TUG, knee extensor strength on the involved leg, T30sSLS on the (most) involved leg, and sleep problem; for (ii) activity on 6MWT and TUG; and for (iii) participation on EUROHIS-QoL and moderate-intensity PADL (Fig. 1, Table 3).

### Clustering on ICF dimensions

For measures with at least moderate effect size, we display the clustering on ICF dimensions in the left column of Table 3 (largest to smallest effect size within each level). However, some measures captured more than one dimension: The TUG mobility and 6MWT captured *(i) the body function* and *(ii) activity* dimensions*,* the Örebro-psychosocial *(i)* and *(iii) the participation* dimension*,* whereas the KOOS and EUROHIS-QoL captured all dimensions *(i-iii)*.

## Discussion

### Principle findings

The main objective of the current study was to explore which recommended/applied measures that most clearly distinguished patients with KOA from healthy controls and describe which ICF dimension those would cluster on. Across 27 measures, the current results indicated that those from the disease specific KOOS (all subscales) and the psychosocial-Örebro questionnaires demonstrated the largest effect sizes for between-group differences, and that these measures together clustered across climbing and the amount of time spent in vigorous-intensity physical activity demonstrated the second largest effect size. These clustered on the ICF dimensions activity and participation, respectively. Finally, sleep problems, knee extension strength, static one-leg balance, endurance walking, and Timed up-and-go all showed moderate effect sizes, where the three first clustered on the body function dimension, and the two latter also clustered on the ICF activity dimension.

### Results discussion

#### ICF body function dimension and KOA

On the ICF body function dimension, our two measures of pain and the KOOS-Symptom indicated very large effect sizes. This is in accordance with findings from patients awaiting total knee replacement [65]. Further, knee pain is the cardinal complaint in KOA and knee symptoms are central in the diagnostic threes of EULAR [6] and ACR [7]. The result of the current study highlights the importance of a proper baseline pain status, in concordance with the importance of monitoring pain within and between strength exercise sessions shown in methodologically and clinically strong RCTs [66–69]. According to recent systematically reviewed trial-evidence [20, 69], pain monitoring is of high value within and between strength-training sessions indicated by the most effective treatment protocols in RCTs also exhibiting high methodological quality [66–69].

The current result indicated a medium between-group difference on knee extension strength. This is comparable to the estimated effect sizes obtained in a meta-analysis of previous cross-sectional case-control studies [*N* = 6086] [70]. Thus, there is ample evidence for patients with KOA demonstrating moderate sized [58] knee extensor weakness compared to healthy controls. Notwithstanding knee extension strength’s medium discrimination, the therapeutic importance of knee extensor strengthening is definitely supported by the Ottawa Panel’s *strongly recommended* strength exercise programs (based on a systematic review of RCTs) [69], wherein large effect sizes [58] of treatment have been found for reducing pain and improving function, in particular after three programs of sitting or lying single-leg strength training [66–68]. These three strength programs showed roughly twice the effect sizes [20, 71] of comparable recommended programs [72], low risk of bias [20], and PEDro-score ≥ 6 of 10. Further, their strengthening dose ([repetitions × resistance × sets]/muscle group) [73] was body-weight independent and objectively recorded [74]. Most importantly though, they coupled pain to dose-response, i.e. linked strength gain to control on pain and objective strengthening dose. Specifically, only these RCTs [66–68] monitored the 24 h load-pain tolerance in a way similar to that explained for treatment of patients with chronic patellar tendinopathy [75]. Further underpinning the importance of pain vs. dose control, OARSI recommends that strength-training logs also include pain levels [76]. Thus, evidence indicates that knee extensor strength has moderate discrimination whereas the 24 h load-tolerance measure shows a promising link to large effects on pain, function, and strength of strength exercise therapy.

The six-minute walk test captures important endurance or long-term walking capability. A relevant question is whether walking only 20 or 40 m, as recommended by ORASI [76], captures the same construct. The present study, however, measured endurance capacity through a six-minute walking test. Herein, the differences between patients with KOA and controls amounted to a moderate sized effect. Our finding is comparable to data from two small-sampled case-control studies on patients awaiting total knee replacement [77, 78]. All these findings are, however, superseded by the large effect size in a much larger case-control study of patients with moderate KOA (*N* = 146) [79]. This contrast to the latter study is understandable, though, given its 24% between-group difference in body mass index (vs. our 3%, 13% [77], 12% [78]). In either case, a meta-analysis of clinical trials has just indicated large effects of aerobic endurance training on pain and physical performance [as compared to usual care] [80], and another such analysis indicated clinically relevant effects on physical function of endurance walking applied as a sole intervention [81]. Thus, evidence indicates at least moderate discrimination of the six-minute walk test and that it offers therapeutically important endurance information for effective endurance therapy in patients with KOA.

#### ICF activity dimension and KOA

Viewed from the activity dimension of ICF, the present findings on KOOS-ADL and KOOS-Sports/Rec were in concordance with the very large effect size seen in patients awaiting total knee replacement [65] and people with radiographic KOA [82] in case-control studies. Further, in a recent meta-analysis of RCTs [80], strength training and mind-body exercises exhibited the largest therapeutic effects on KOOS-ADL and WOMAC physical function relative to other active therapies that were also compared to standard care. Furthermore, a RCT documented better outcomes when the intervention was a similar (i.e., disease-specific) questionnaire used as a checklist as compared to usual care [83]. Moreover, the above-mentioned trial meta-analysis [80] also indicated large therapeutic effects on pain and physical performance of intensive aerobic endurance exercises. Importantly, such exercises are captured in the problematic activity of the KOOS-Sports/Rec. Thus, evidence indicates important discrimination on KOOS-ADL and KOOS-Sport/Rec, important outcome-measure value of the KOOS-ADL, and finally, that the KOOS-Sports/Rec needs further evaluation as an outcome measure in KOA.

The up-and-down stair climb test demonstrated a large between-group difference in the current study. This is comparable to the 160% longer average climbing-time for patients with KOA in another similar study [78]. However, the point-estimated effect size in the latter only reached a moderate magnitude, as did the finding in another large case-control study [79]. Indeed, similar-numbered up-and-down stairs climb tests have shown problems of both validity and reliability in musculoskeletal [47] and KOA populations [40]. Increasing and fixing the time of walking upwards to 20 s and rather record the numbers of negotiated steps thus seems like a promising alternative [40]. Further, stair negotiation as a sole exercise therapy has documented very weak evidence and indeterminable outcomes in a systematic review of RCTs for patients with total knee replacement [84]. Thus, the evidence indicates medium discrimination of a 10 stairs up-and-down climb test with an uncertain connection to effective therapy.

#### ICF participation dimension and KOA

On the ICF participation dimension in the present study, the KOOS Quality of life measure amounted to a very large effect size, which is concurrent with that reported in two previous case-control studies [78, 82]. Further, meta-analyses of RCTs indicate important effects of exercise therapy [85] and strength training [80] on health- and knee-related quality of life. Therefore, evidence indicates that the KOOS quality of life measure has very large discriminative value and important outcome measure value in effective trials for patients with KOA.

The current study found a very large between-group difference on the Örebro-psychosocial measure. Surprisingly, no previous case-control studies on KOA appear to have used this questionnaire. Further, according to a systematic review [86], therapy for psychosocial factors might only be of limited additive importance for patients with KOA because no significant effect of psychotherapy on pain was documented. The latter is concurrent with our patients’ mild score on kinesiophobia. For the individual patient, however, those scoring above 60% on the Örebro-psychosocial questionnaire have shown to be of high risk for absenteeism [work/social activities] [87]. Therefore, more studies are needed to challenge the present discriminative ability and evaluate the therapeutic relevance of the Örebro-psychosocial measure in KOA.

When looking for relevant case-control studies to compare the large between-group difference in vigorous-intensity physical activity of the present study, we did not find any. Interestingly though, when compared to the 2018 American physical activity guideline for adults [88] which recommends at least 75 min to 150 min of vigorous-intensity aerobic physical activity a week, both patients and controls were far from the target (average 0 vs 29 min). More relevant perhaps, the alternative of the same guideline is 150 to 300 min of moderate-intensity physical activity a week, whereupon our patients were seemingly on the target (mean 286 min), although the variability amongst them was substantial (SD of 169 min). Our moderate between-group difference in moderate-intensity physical activity, however, agreed with that reported in two such previous studies [89, 90]. Thus, evidence indicates that objectively obtained levels of moderate-intensive physical activity have moderate discrimination and obvious/inherent outcome measure value, but that its therapeutic value needs evaluation in KOA.

### Possible implications

For implications, the most important health measures are those that offer important information on diagnosis/situational understanding, prognosis/therapy, and outcome-evaluation, while being reasonably fast, easily applicable, and low cost. Most of our measures with at least moderate effect sizes might be applicable for such purposes [6, 91]. More importantly, in several case-control studies, the KOOS has shown very large effect sizes on most factors across all ICF dimensions. Although the KOOS is highly discriminative, cost-free, and disease-specific [18, 35], it does not collect frequency and intensity of activities in contrast to for example the generic University of California at Los Angeles activity rating scale [92], the International Physical Activity Questionnaire [93], and the Frenchay Activities Index [35, 94]. For clinicians and researchers, these complementary patient-reported measures are easily available and cost-free [35] or available in web-based computer systems at non-profitable costs (e.g. InfoPad [28] and PROMIS [95]).

Importantly, because only some of the present measures showed substantial discrimination and clear links to effective therapy, we hope the discussion-adjusted conclusions [96] herein raise priority-concerns about which ones to apply clinically or evaluate further in exploratory and confirmative studies [24].

### Methods discussion

The current study has its limitations and strengths. On the one hand, we did not manage to match the groups on age, nor was it possible to adjust for its potential age-inflating functional decline effects on the non-parametric data. Further, the current sample size can be considered rather small if one assumes that adjustments were needed for multiple comparisons in this study according to classical statistical texts [54, 97–100]. On the other hand, the age-adjusted sensitivity analysis of the parametric data supported the unadjusted analysis. Further, both age-means were well within the same clinical middle-aged maturation category (Mesh, PubMed). Yet further, the Results discussion (of the current study) revealed current results in general agreement with those of optimally age-matched confirmative studies. Moreover, for the therein present “inflated” (stair climbing) or “deflated” (endurance walking) results, true result variation among samples [54, 97] is perfectly normal also among low risk-of-bias studies (sifted in meta-analyses) [70]. Even further, according to reputative statisticians, there was no need [22–27], or inappropriate or even deleterious to sound statistical inferences [22, 23, 27], to correct for multiple comparisons in the present exploratory study. Additionally, according to the current sample size calculation and the 27 tests, the indicated *number* of false positive results (type I errors) was 27 × 0.05 = 1.35 [24]. Or, just one of our significant results was most certainly false. Should we thus have adjusted for multiple comparisons? We believe not, because the assumption of such adjustments is the “universal null hypothesis” that holds random variation as the first-order explanation, thus undercutting the premises that nature follows regular laws [22]. And because, in the present study, making no such adjustments kept the statistical power high and type II error-rate low [22, 27]. Concordantly, in the present exploratory low-risk-measures’ study, we cared *way more* about finding true differences than being afraid of accepting a false positive finding. Thus, we infer reasonable internal validity of this study.

The current study weighed its external validity against important considerations. Small-sampled studies [e.g., *n* < 20] [54, 97] are known to increase the risk of chance inflated/deflated effects and thus decrease the generalizability compared to large-sampled studies [e.g., *n* > 100] [54, 97], whereas both too small or too large samples are unacceptable for clinical, methodological, and ethical reasons [101]. Comparingly, the current study’s sample-size was moderate [e.g., *n* ≥ 20 ≤ 100] [54, 97], minimalized according to calculated requirements [with advantages on cost, feasibility, and patients’ burden] [101], powered higher than comparable exploratory studies [102, 103], and aligned with the assumption of no need for adjustments of multiple comparisons in exploratory designs [22–27]. Admittingly, the current study limited its generalizability to patients aged < 70 and with BMI < 35. The upper age-limit was sat mainly due to the risk of higher comorbidity in the KOA-group at higher ages [32], whereas the upper limit on BMI (including WHO’s obesity class I, excluding class II-III) was sat to preserve the repeatability of collected optoelectronic kinematics [34] (data not published here). Comparably, more than 3900 patients with KOA in over 50 Cochrane-reviewed trials were dominated by the middle-aged and aged maturation categories (45–70 years old) with a mean BMI ranging 25 to 32 [72]. Thus, even though an uncertainty remains due to the partly unadjusted age-difference, the external validity of our findings seems substantial. The largest strength of the present study is to show the quantified rank of a plethora of recommended and applied measures in KOA [17–20] whereof only a minority showed considerable between-group differences. The current findings presumably prompt important priority concerns. However, such concerns should at least be influenced by the effects of therapy on these measures (cf. the Results discussion above).

## Conclusions

In conclusion, among 27 relevant measures, this present study indicates very large differences across all ICF dimensions for the Knee Injury and Osteoarthritis Outcome Scale (KOOS) and the Örebro-psychosocial questionnaire (OMSPQ-10) in patients aged 45–70 with mild to moderate KOA in a primary/hospital care setting. Clinicians might consider screening by these instruments as means of selecting among relevant supplementary measures demonstrating appropriate discriminative characteristics and clear links to effective therapy. Confirmative studies are needed to further validate these explorative and partly age-unadjusted conclusions.

## Supplementary information

**Additional file 1.** Additional methods information regarding measurement instruments, data processing, and statistics applied in Vaarbakken et al. (2019).

## Data Availability

The data will be available by reasonable request made to head of project A. K. Stensdotter or to the project owner NTNU, Department of Neuromedicine and Movement Science (INB), Program for Physiotherapy.

## Abbreviations

6MWT: six-minute walk distance test;
AB: Aktiebolag, Swedish for a limited liability company
ACR: American College of Rheumatology
ADL: Activities in daily living
ANCOVA: Analysis of co-variance
AX3 3D accelerometer: A three dimensional or axed accelerometer with the brand name AX3 3D
EULAR: European League Against Rheumatism
EUROHIS-QoL: European Health Interview Survey-Quality of Life 8-item index
GP: general physician
ICF: International Classification of Function, Disability, and Health
KOA: Knee Osteoarthritis
KOOS: Knee Injury and Osteoarthritis Outcome Scale
N: Total number of participants in the meta-analyzed studies or in a single case-control study
n30sCST: number of stands in the 30 s Chair to Stand Test
NPRS: Numeric Pain Rating Scale
OARSI: Osteoarthritis Research Society International
OMSPQ: Örebro Musculoskeletal Pain Screening Questionnaire
OMSPQ-10: Örebro Musculoskeletal Pain Screening Questionnaire 10 item version
PADL: physical activities in daily life
PSFS: Patient specific functional scale
QoL: Knee-related quality of life (in KOOS) or generic quality of life (in the EUROHIS-QoL instrument)
QTM: Qualisys Track Manager
REC: Regional Ethics Committee for Medical and Health Research
RPE-CR10: Borg’s Rating of Perceived Exertion Category Ratio 10
SMD: Standardized mean difference
Sports/Rec: Sport and recreation (part of KOOS)
SPSS: Statistical Package for the Social Sciences, the original name of a statistical software today used in various research areas
SROM: self-reporting outcome measure
T10StUpDw: Timed 10-step up-and-down stair climb test
T30sSLS: Timed maximum 30 s single-leg stance test
TSK-13: Tampa Scale of Kinesiophobia 13 item version
TUG: Timed up-and-go
WOMAC: Western Ontario and McMaster Universities Osteoarthritis Index
