# Supplementary material for: Relative difference among 27 functional measures in patients with knee osteoarthritis: an exploratory cross-sectional case-control study
Source: BMC Musculoskelet Disord. 2019 Oct 22;20:462. doi: 10.1186/s12891-019-2845-0 (PMC6805424; doi:10.1186/s12891-019-2845-0)
Supplement: Supplementary file 1 — Additional file 1. Additional methods information regarding measurement instruments, data processing, and statistics applied in Vaarbakken et al. (2019). [file 12891_2019_2845_MOESM1_ESM.docx]

**Additional file 1.** Additional methods information regarding measurement instruments, data processing, and statistics applied in Vaarbakken et al. (2019).

[Note that this additional file 1 has its own reference list below.]

## Measurement instruments

#### Body function measures

**Strength**: The Biodex System 4 dynamometer (Biodex Medical Systems Inc., NY, USA) produced similar values as the Cybex dynamometer for maximum knee extensor strength in concentric mode at 60°/s in healthy male adults (1). Herein, high-to-very-high reproducibility for concentric peak strength (ICC 0.92) with small difference between dynamometers (SEM 3.7 Nm; CV 5.3%). In healthy people mean aged 71 years, the test-retest ICCs of the isokinetic quadriceps strength were high (0.81-0.99), indicating good to very good reliability, and most SEM and rate of limit of agreement indexes indicated acceptable agreement (2).

**The 6 Minute Walk distance Test** (6MWT) is an objective measure of sub-maximal aerobic capacity and walking ability. In a psychometric study of individuals with KOA, pooled mean [SD] was 548.2 [86.6] m (3). Between-rater and within-rater reliability were good [relative ICC 0.94, 0.93] and absolute reliability adequate [standard error of measurement (SEM) 21.3m (3.9%), 18.1m (3.3%)] (3). In individuals after total knee replacement (TKR), absolute reliability by SEM of 26.3 m, minimal detectable change (MDC) of 61.3 m, and excellent test-retest reliability (ICC 0.94) were documented (4). Further, minimal clinical important difference (MCID) was 26-55 m for mobility 26 weeks post TKR (5). For elderly individuals, MDC of 20 m and MCID of 50 m were documented (6).

**30 seconds Chair to Stand Test** (n30sCST) [n = number of stands]: It assesses functional lower extremity strength or fitness (3). In a psychometric study of individuals with KOA, mean (SD) of has been 11.6 (2.7) number of stands (3). It has proven good between-rater reliability, adequate size of measurement error (3) and responsiveness in individuals with KOA to post TKR (7) and MCID in individuals with HOA of ≥ 2.6 repetitions (8).

**Timed max 30sec single-leg stance** (T30sSLS): It tests the static balance of individuals in a sensory integration taxing condition (9). It has documented good between-rater reliability and within-rater reliability for individuals with hip osteoarthritis (HOA) (10). Further, good test-retest and between-rater reliability for healthy adult women with eyes open (11). Convergent validity was r = 0.71 with the Community Balance and Mobility Scale (12). Normative data for a healthy mixed population ranged 16-37 were mean (SD) 20 (3) seconds (13). In the present study, we recorded the time for single leg standing by two force plates (Kistler, NY, USA).

**Numeric Pain Rating Scale (NPRS)** is a unidimensional scale, 0-10, best to worst. In a psychometric study of individuals with KOA, mean (SD) was 5.3 (1.8) for average knee pain last week (3). It has shown good reliability (ICC 0.89), small measurement error (SEM 0.71 point) and MCD of 1.96 points in individuals with KOA (14). Further, MCID of 1 to 4 points (15-28%) in mixed chronic pain populations including KOA (15-17). Our study applied the time frame ‘on average the last week’.

**Sleep and Vitality:** Self-reported Sleep quality and Vitality were both registered by a 1 to 5 custom made Likert scale. For Sleep, 1) “I sleep normally, wakens afresh, and have no problem sleeping”, 2) “I have mild sleep problems, f. ex. difficulty falling asleep or wake up sometimes at night”, 3) “I have moderate sleep problems, f. ex. disturbed sleep or feel that I havn’t slept enough”, 4) “I have severe sleep problems, f. ex. need sleep medication often or regularly, or awakens at night and or too early in the morning”, and 5) “I suffer from serious insomnia”. For Vitality, 1) “I feel healthy and energetic”, 2) “I feel mildly tired, sleepy, or weak”, 3) “I feel moderately tired, sleepy, or weak”, 4) “I feel extremely tired, sleepy, or weak, almost exhausted”, 5) “I feel extremely tired, sleepy or weak, totally exhausted”, respectively.

**KOOS-Pain**: In methodological studies of individuals with KOA, the pooled mean [SD] score was 51 [18] (18). Construct validity, correlation with Short Form 36 (SF-36) pain, physical function, role-emotional, mental health, were reasonable for individuals with KOA (18). Internal consistency was good (Cronbach's α 0.9) and SEM 9.5 (18).

**KOOS-symptoms:** In methodological studies of individuals with KOA, the pooled mean [SD] score was 52.9 [18.4] (18). For construct validity, correlation with Short Form 36 (SF-36) pain, physical function, role-emotional, and mental health, were reasonable (18). Further, internal consistency was good (by Cronbach's α 0.87), SEM 7.6, and SDC 21.2.

**Kinesiophobia**, or debilitating fear of movement, was assessed using 13 of 17 questions of the Tampa Scale of Kinesiophobia (TSK) that is found adequately reliable and valid in individuals with low back pain (19, 20). The TSK-13 scores 13 to 52, best to worst, where subclinical range is suggested as 13 to 22 points, mild as 23 to 32, moderate as 33 to 42, and severe as 43 to 52 (21).

**Borg’s Rating of Perceived Exertion, Category Ratio 10** (RPE-CR10), scaled 0 to 10, nothing at all to extremely strong (22), is widely used to subjectively quantify the perception of the physical demands of an activity. Originally validated against heart rate (r = 0.80–0.90), later extensively validated (23-25), f. ex. in individuals with fibromyalgia (26) and recovering athletes (27). This measure was used together with some performance tests (as specified in the main paper).

**Endurance** was evaluated by 6MWT and the RPE-CR10 (see 6MWT under Activity).

Within the ICF, we classified the n30sCST to the body function level, because it doesn’t include “the ability to move around” (28) and “the ability to perform daily activities” (29). This in contrast to for example Dobson et al. (2017), whom classified it as an activity (3).

#### Activity measures

**Timed 9-12 step up and down stair climb test** (T10StUpDw or T10SCT): In a systematic review, Dobson et al. (30) reported the following: “Evidence for stair negotiation tests [and other performance tests] was limited by small sample sizes or inappropriate time intervals between repeated testing.” (p. 1555). They concluded: “Only evidence of construct validity was reported for the 12 step stair test for knee OA. Given the current limited evidence of stair negotiation tests, recommendations about which tests might be more useful cannot be made.” (p. 1558). However, for the SCT performed as timed up and down 9 stairs in 21 individuals awaiting TKR/THR, Kennedy et al. (4) documented the following: Preoperative time mean (SD) 17.1 (8.2) seconds, responsiveness by SRM pre-post (95% CI) of -1.74 (-2.13, -1.45) seconds, absolute reliability as SEM (95% CI) 2.35 (1.89, 3.10) seconds, MDC_90_ of 5.49 seconds, and excellent reliability by ICC (95% CI) 0.90 (0.79, 0.96). Their test-retest interval was 91 days. Further, for a SCT performed as up and down 12 step test in 100 individuals awaiting TKR, Mizner et al. (31) supported its construct validity by moderate negative correlation with knee extension strength (r = −0.46, highly significant) and poor correlation with a PROM, both as predicted. They also reported that one-month post TKR, the order of responsiveness, from largest to smallest effect size of change (either improvement or worsening), respectively, was SCT, 6MWT, TUG, Knee Outcome Survey–Activities of Daily Living Scale. Moreover, for a timed 9 step up and down SCT in 25 individuals with KOA from the community, Rejeski et al. (32) documented excellent test-retest reliability coefficient (r = 0.93), given a test-retest interval of 14 days. Herein, construct validity was supported by moderate positive correlation with the patient-reported outcome measure (PROM) Functional' Performance Inventory for ambulation/climbing (FAST) [unadjusted (adjusted) 0.48 (0.35)], and by negative correlation with knee extension strength [unadjusted (adjusted) -0.51 (-0.20)]. However, in support of the view given in the systematic review by Dobson et al (2012), is a study (3) on a timed-11-step-up-and-down SCT in 50 individuals with moderate KOA-and-HOA. Here Dobson et al. (2017) documented that their SCT did not meet their preset minimal acceptable level for relative between-rater reliability or within-rater reliability [ICC 0.78, lower 1-sided 95% CI 0.67; 0.78, 0.50), respectively]. Their absolute reliability was SEM of 1.00 or 8% (95% CI 0.83, 1.24). Their test-retest interval was one week. In sum, however, evidence indicate reasonable measurement properties for a timed 9-12 step up and down stair climbing test for individuals with moderate to severe KOA in adults.

**The Timed up and go (TUG)** assesses mobility, balance, walking ability, and fall risk (9). In individuals with KOA, the mean (SD) was 8.4 (1.4) seconds (3). It demonstrated high within-rater and between-rater reliability (ICC 0.97, 0.96), low SEM (0.16 sec or 8.0%), MDC of 1.10 sec and 1.14 sec, respectively (3, 33). In people with TKR, the test-retest reliability was fair (ICC 0.75) and standardized response mean (SRM) 1.04 seconds (4). Convergent validity with Community Balance and Mobility Scale was r -0.74 (12). In HOA, the MCID after physiotherapy was of 0.8 to 1.4 seconds (8, 34).

**KOOS-ADL:** In psychometric studies, its mean (SD) score was 54.2 (18.6) for individuals with KOA (18), construct validity correlated sensibly with Short Form 36 (SF-36) pain, physical function, role-emotional, mental health (18). Internal consistency was good (Cronbach's α 0.89), SEM 8.6, and SDC averaged 24 points (18).

**KOOS-Sports/Rec:** In KOA individuals, its mean [SD] score was 21.9 [24.9] (18). For construct validity sensible correlation with Short Form 36 (SF-36) pain, physical function, role-emotional, and mental health were documented in individuals with KOA (18). Further, internal consistency was adequate (Cronbach's α 0.83), SEM 17.7, and smallest detectable difference (SDC) 49.1.

According to a systematic review, users of KOOS can be confident of content validity, internal consistency, test-retest reliability, construct validity and responsiveness for age- and condition-relevant subscales (18).

**Patient specific functional scale (PSFS)**: For individuals with knee dysfunction, the sensitivity to change was adequate (ICC 0.77), test-retest reliability good, and SEM 1.0 point [90% CI MDC 2.5 points] (35). In patients with TKR, the SRM at 3 months and 1 year were 0.96 and 1.48 points, respectively (36). These authors concluded the reliability of the PSFS was good and the responsiveness high.

**Practical application of performance tests**

A description of each of the OARSI recommended performance based test (30sCST, 40mFPWT [not assessed in the present study], SCT [i.e., a 11 Steps Up and Down Climb test], TUG and 6MWT) including set up, equipment, preparation (environment, participant, and tester), procedures, verbal instructions and scoring are available on the OARSI website: <http://oarsi.org/research/physical-performancemeasures>.

#### Participation measures

**KOOS-QoL:** In individuals with KOA the mean [SD] score was 35.1 [19.2] (18). For construct validity, sensible correlation with Short Form 36 (SF-36) pain, physical function, role-emotional, and mental health were documented (18). Internal consistency by pooled Cronbach's α was 0.84, SEM 9 points, and finally SDC 24.9.

**The European Health Interview Survey-Quality of Life 8-item index** **[EUROHIS-QoL]** (37) is an free generic QoL-measure developed from the WHOQoL-BREF (38), again developed from the WHOQoL 100-item questionnaire (39). KV reduced the Norwegian WHOQOL-BREF (40) into the EUROHIS-QoL 8-item index. Its scale is 8-40 points, worst to best. In individuals after hip and knee replacement, EUROHIS-QoL 8 showed high internal consistency (α = 0.86), adequate convergent validity against WOMAC (r = 0.47-0.82), and adequate discriminant validity (41). Further, it showed adequate properties for evaluating general health and function and psychosocial aspects of QoL, as well as minimal ceiling effects (41). And it has shown good cross-cultural performance and satisfactory convergent and discriminant validity across 10 European countries (37).

**The Örebro Musculoskeletal Pain Screening Questionnaire** (OMSPQ) was developed as a tool to identify yellow flags and patients in risk of work disability due to pain (42, 43). The OMSPQ has 25 items with satisfactory psychometric properties and predictive ability (44). More recently, a short form of the OMPSQ (OMPSQ-10) has been developed, improving its clinical utility due to its shorter length and similar psychometric properties in a mixed musculoskeletal population (45). OMPSQ-10’s scale is 1-100, best to worst.

**Activity intensity logging:** The AX3 3D accelerometer sensor (Axivity, Newcastle, UK) is sized 23×32.5×7.6 mm and weights 11 g. AX3 was the most accurate accelerometer during walking compared to two similar sensors recommended for such analysis (46). Further, when placed on the thigh and upper back, and sampled at ~100 Hz, it was highly valid against video recordings in a lab environment (47). When a single thigh accelerometer was used, as in the present study, the accuracy of walking and running activities remained stable (47).

### Data processing

#### Cutpoints for ADL-intensities from accelerometers

The cutpoints for the four activity intensity levels were: sedentary < 1.5 Metabolic Equivalents of Tasks (METs), light > 1.5 < 3.99, moderate > 3.99 to < 6.99, and vigorous > 6.99. Data were given as times in specific intensities of physical activity (PA). That is, in relation to the rate of energy produced per unit surface area of an average person seated at rest (1 MET = 3.5 ml O^2^×kg^-1^×min^-1^, or 58.2 W/m^2^).

### Statistics

Hopkins’ scale (48) of interpretation of effect sizes of Cohen’s d (trivial < 0.2, small ≥ 0.2, moderate ≥ 0.6, large ≥ 1.2, very large ≥ 2.0, near perfect ≥ 4.0, perfect = infinite). Rosner’s scale (49) for interpreting p-values of hypothesis tests: The If: .01 ≤ p < .05 significant, .001 ≤ p < .01 highly significant, .0001 ≤ p < .001 very highly significant, and if p < .0001 extremely highly significant. If p > .05, then the results are considered not statistically significant.

### Test team and supervisors

The test team consisted of a phd-student physiotherapist with 4 years of clinical experience (KV), two master students of movement science (EBK, AL), and a bachelor student of physiotherapy (TMBM). The team had two experienced supervisors (AKS and DR, see Acknowledgement).

**Reference List**

1. de Araujo Ribeiro Alvares JB, Rodrigues R, de Azevedo Franke R, da Silva BG, Pinto RS, Vaz MA, et al. Inter-machine reliability of the Biodex and Cybex isokinetic dynamometers for knee flexor/extensor isometric, concentric and eccentric tests. Physical therapy in sport : official journal of the Association of Chartered Physiotherapists in Sports Medicine. 2015;16(1):59-65.

2. Hartmann A, Knols R, Murer K, de Bruin ED. Reproducibility of an isokinetic strength-testing protocol of the knee and ankle in older adults. Gerontology. 2009;55(3):259-68.

3. Dobson F, Hinman RS, Hall M, Marshall CJ, Sayer T, Anderson C, et al. Reliability and measurement error of the Osteoarthritis Research Society International (OARSI) recommended performance-based tests of physical function in people with hip and knee osteoarthritis. Osteoarthritis Cartilage. 2017;25(11):1792-6.

4. Kennedy DM, Stratford PW, Wessel J, Gollish JD, Penney D. Assessing stability and change of four performance measures: a longitudinal study evaluating outcome following total hip and knee arthroplasty. BMC musculoskeletal disorders. 2005;6:3.

5. Naylor JM, Mills K, Buhagiar M, Fortunato R, Wright R. Minimal important improvement thresholds for the six-minute walk test in a knee arthroplasty cohort: triangulation of anchor- and distribution-based methods. BMC musculoskeletal disorders. 2016;17(1):390.

6. Perera S, Mody SH, Woodman RC, Studenski SA. Meaningful change and responsiveness in common physical performance measures in older adults. Journal of the American Geriatrics Society. 2006;54(5):743-9.

7. Gill SD, de Morton NA, Mc Burney H. An investigation of the validity of six measures of physical function in people awaiting joint replacement surgery of the hip or knee. Clinical rehabilitation. 2012;26(10):945-51.

8. Wright AA, Cook CE, Baxter GD, Dockerty JD, Abbott JH. A comparison of 3 methodological approaches to defining major clinically important improvement of 4 performance measures in patients with hip osteoarthritis. The Journal of orthopaedic and sports physical therapy. 2011;41(5):319-27.

9. AbilityLab SR. Rehabilitation Measures Database 2018 [Available from: <https://www.sralab.org/rehabilitation-measures>

10. Choi YM, Dobson F, Martin J, Bennell KL, Hinman RS. Interrater and intrarater reliability of common clinical standing balance tests for people with hip osteoarthritis. Phys Ther. 2014;94(5):696-704.

11. Franchignoni F, Tesio L, Martino MT, Ricupero C. Reliability of four simple, quantitative tests of balance and mobility in healthy elderly females. Aging (Milan, Italy). 1998;10(1):26-31.

12. Takacs J, Garland SJ, Carpenter MG, Hunt MA. Validity and reliability of the community balance and mobility scale in individuals with knee osteoarthritis. Phys Ther. 2014;94(6):866-74.

13. Schneiders AG, Sullivan SJ, Gray AR, Hammond-Tooke GD, McCrory PR. Normative values for three clinical measures of motor performance used in the neurological assessment of sports concussion. Journal of science and medicine in sport / Sports Medicine Australia. 2010;13(2):196-201.

14. Alghadir AH, Anwer S, Iqbal ZA. The psychometric properties of an Arabic numeric pain rating scale for measuring osteoarthritis knee pain. Disability and rehabilitation. 2016;38(24):2392-7.

15. Cimmino MA, Salaffi F, Olivieri I, Trotta F, Frizziero L, Sarzi Puttini P, et al. [Pain patterns in Italian patients with osteoarthritis: preliminary results of the MI.D.A. Study (Misurazione del Dolore nell'Artrosi)]. Reumatismo. 2004;56(4):253-61.

16. Goldsmith ES, Taylor BC, Greer N, Murdoch M, MacDonald R, McKenzie L, et al. Focused Evidence Review: Psychometric Properties of Patient-Reported Outcome Measures for Chronic Musculoskeletal Pain. Journal of general internal medicine. 2018;33(Suppl 1):61-70.

17. Farrar JT, Young JP, Jr., LaMoreaux L, Werth JL, Poole RM. Clinical importance of changes in chronic pain intensity measured on an 11-point numerical pain rating scale. Pain. 2001;94(2):149-58.

18. Collins NJ, Prinsen CA, Christensen R, Bartels EM, Terwee CB, Roos EM. Knee Injury and Osteoarthritis Outcome Score (KOOS): systematic review and meta-analysis of measurement properties. Osteoarthritis Cartilage. 2016.

19. Haugen AJ, Grovle L, Keller A, Grotle M. Cross-cultural adaptation and validation of the Norwegian version of the Tampa scale for kinesiophobia. Spine (Phila Pa 1976). 2008;33(17):E595-601.

20. Damsgard E, Fors T, Anke A, Roe C. The Tampa Scale of Kinesiophobia: A Rasch analysis of its properties in subjects with low back and more widespread pain. Journal of rehabilitation medicine. 2007;39(9):672-8.

21. Neblett R, Hartzell MM, Mayer TG, Bradford EM, Gatchel RJ. Establishing clinically meaningful severity levels for the Tampa Scale for Kinesiophobia (TSK-13). European journal of pain (London, England). 2016;20(5):701-10.

22. Borg G. Borg's perceived exertion and pain scales. Champaign, Ill. Human Kinetics1998.

23. Pescatello LS, Arena R, Riebe D, Thompson PD. ACSM's guidelines for exercise testing and prescription. 9th ed. ed. Philadelphia, Pa: Wolters Kluwer Health/Lippincott Williams & Wilkins; 2014.

24. Chen MJ, Fan X, Moe ST. Criterion-related validity of the Borg ratings of perceived exertion scale in healthy individuals: a meta-analysis. Journal of sports sciences. 2002;20(11):873-99.

25. Ritchie C. Rating of perceived exertion (RPE). Journal of physiotherapy. 2012;58(1):62.

26. Newcomb LW, Koltyn KF, Morgan WP, Cook DB. Influence of preferred versus prescribed exercise on pain in fibromyalgia. Medicine and science in sports and exercise. 2011;43(6):1106-13.

27. Hamer P, Slocombe B. The psychophysical and heart rate relationship between treadmill and deep-water running. Aust J Physiother. 1997;43(4):265-71.

28. Bellamy N, Kirwan J, Boers M, Brooks P, Strand V, Tugwell P, et al. Recommendations for a core set of outcome measures for future phase III clinical trials in knee, hip, and hand osteoarthritis. Consensus development at OMERACT III. The Journal of rheumatology. 1997;24(4):799-802.

29. Terwee CB, Mokkink LB, Steultjens MP, Dekker J. Performance-based methods for measuring the physical function of patients with osteoarthritis of the hip or knee: a systematic review of measurement properties. Rheumatology(Oxford). 2006;45(7):890-902.

30. Dobson F, Hinman RS, Hall M, Terwee CB, Roos EM, Bennell KL. Measurement properties of performance-based measures to assess physical function in hip and knee osteoarthritis: a systematic review. Osteoarthritis Cartilage. 2012;20(12):1548-62.

31. Mizner RL, Petterson SC, Clements KE, Zeni JA, Jr., Irrgang JJ, Snyder-Mackler L. Measuring functional improvement after total knee arthroplasty requires both performance-based and patient-report assessments: a longitudinal analysis of outcomes. The Journal of arthroplasty. 2011;26(5):728-37.

32. Rejeski WJ, Ettinger WH, Jr., Schumaker S, James P, Burns R, Elam JT. Assessing performance-related disability in patients with knee osteoarthritis. Osteoarthritis Cartilage. 1995;3(3):157-67.

33. Alghadir A, Anwer S, Brismee JM. The reliability and minimal detectable change of Timed Up and Go test in individuals with grade 1-3 knee osteoarthritis. BMC musculoskeletal disorders. 2015;16:174.

34. Dobson F. Timed Up and Go test in musculoskeletal conditions. Journal of physiotherapy. 2015;61(1):47.

35. Chatman AB, Hyams SP, Neel JM, Binkley JM, Stratford PW, Schomberg A, et al. The Patient-Specific Functional Scale: measurement properties in patients with knee dysfunction. Phys Ther. 1997;77(8):820-9.

36. Berghmans DD, Lenssen AF, van Rhijn LW, de Bie RA. The Patient-Specific Functional Scale: Its Reliability and Responsiveness in Patients Undergoing a Total Knee Arthroplasty. The Journal of orthopaedic and sports physical therapy. 2015;45(7):550-6.

37. Schmidt S, Muhlan H, Power M. The EUROHIS-QOL 8-item index: psychometric results of a cross-cultural field study. European journal of public health. 2006;16(4):420-8.

38. Skevington SM, Lotfy M, O'Connell KA. The World Health Organization's WHOQOL-BREF quality of life assessment: psychometric properties and results of the international field trial. A report from the WHOQOL group. Quality of life research : an international journal of quality of life aspects of treatment, care and rehabilitation. 2004;13(2):299-310.

39. Group TW. The World Health Organization Quality of Life Assessment (WHOQOL): development and general psychometric properties. Social science & medicine (1982). 1998;46(12):1569-85.

40. Kalfoss MH, Isaksen AS, Thuen F, Alve S. The suitability of the World Health Organization Quality of Life Instrument-BREF in cancer relatives. Cancer nursing. 2008;31(1):11-22.

41. Snell DL, Siegert RJ, Surgenor LJ, Dunn JA, Hooper GJ. Evaluating quality of life outcomes following joint replacement: psychometric evaluation of a short form of the WHOQOL-Bref. Quality of life research : an international journal of quality of life aspects of treatment, care and rehabilitation. 2016;25(1):51-61.

42. Linton SJ, Hallden K. Can we screen for problematic back pain? A screening questionnaire for predicting outcome in acute and subacute back pain. The Clinical journal of pain. 1998;14(3):209-15.

43. Linton SJ, Boersma K. Early identification of patients at risk of developing a persistent back problem: the predictive validity of the Orebro Musculoskeletal Pain Questionnaire. The Clinical journal of pain. 2003;19(2):80-6.

44. Hockings RL, McAuley JH, Maher CG. A systematic review of the predictive ability of the Orebro Musculoskeletal Pain Questionnaire. Spine (Phila Pa 1976). 2008;33(15):E494-500.

45. Linton SJ, Nicholas M, MacDonald S. Development of a short form of the Orebro Musculoskeletal Pain Screening Questionnaire. Spine (Phila Pa 1976). 2011;36(22):1891-5.

46. Feng Y, Wong CK, Janeja V, Kuber R, Mentis HM. Comparison of tri-axial accelerometers step-count accuracy in slow walking conditions. Gait Posture. 2017;53:11-6.

47. Stewart T, Narayanan A, Hedayatrad L, Neville J, Mackay L, Duncan S. A Dual-Accelerometer System for Classifying Physical Activity in Children and Adults. Medicine and science in sports and exercise. 2018.

48. Hopkins WG. A Scale of Magnitudes for Effect Statistics 2002 [Available from: <http://www.sportsci.org/resource/stats/effectmag.html>

49. Rosner B. Fundamentals of biostatistics. 8th ed. ed. Boston, Mass: Cengage Learning; 2016.
